# Prognostic role of long non-coding RNA XIST expression in patients with solid tumors: a meta-analysis

**DOI:** 10.1186/s12935-018-0535-x

**Published:** 2018-03-09

**Authors:** Huihui Mao, Kai Wang, Yuehua Feng, Jun Zhang, Lili Pan, Yuxia Zhan, Haijun Sheng, Guanghua Luo

**Affiliations:** 1 Comprehensive Laboratory, Changzhou Key Lab of Individualized Diagnosis and Treatment Associated with High Technology Research, The Third Affiliated Hospital of Soochow University, Changzhou, 213003 China; 2 Department of Urology, The Third Affiliated Hospital of Soochow University, Changzhou, 213003 China

**Keywords:** Long non-coding RNA, XIST, Prognosis, Meta-analysis

## Abstract

**Background:**

The aberrant expression of long non-coding RNA (lncRNA) X inactivate-specific transcript (XIST) has been demonstrated to be involved in the tumourigenesis and the development of various cancers. Therefore, we conducted a meta-analysis to assess the prognostic role of lncRNA XIST expression in solid tumors.

**Methods:**

The databases of PubMed, EMBase, Web of Science, Cochrane library (up to Dec 31, 2017) were searched for the related studies and identified 15 eligible studies containing 1209 patients to include in the meta-analysis. Hazards ratios (HRs) with corresponding 95% confidence intervals (CIs) were pooled to estimate the association between lncRNA XIST expression and survival of cancer patients from Asian.

**Results:**

The result showed that higher lncRNA XIST expression in cancer tissue was related to a worse overall survival (OS) (HR = 1.54, 95% CI 1.07–2.23). In subgroup analysis, it revealed that lncRNA XIST overexpression was significantly associated with worse OS in digestive system tumors (HR = 1.67, 95% CI 1.11–2.51, *p *= 0.031). In addition, the association between high lncRNA XIST expression and poor OS was also statistically significant in other subgroups, including multivariate analysis (HR = 2.39, 95% CI 1.28–4.46, *p *= 0.006, random-effect), patients’ number was greater than 65 (HR = 1.75, 95% CI 1.24–2.47, *p *= 0.001, random-effect), and reported in text (HR = 2.50, 95% CI 1.49–4.18, *p *= 0.000, random-effect).

**Conclusions:**

The expression of lncRNA XIST could be regarded as a poor prognostic biomarker for solid tumors, which might shed new light on epigenetic diagnostics and therapeutics in tumors.

## Background

Both the number of cancer patients and the mortality rate are disturbingly increasing. Cancer has become a common disease that is seriously detrimental to human health, which is a significant cause of death in many countries around the world. Despite the dramatic developments in the diagnosis and therapy of tumors over the past few decades, the overall survival (OS) of patients remains unsatisfactory. Tumor markers play a significant role in monitoring and treating tumors. However, fewer tumor markers were used in clinical diagnosis. Therefore, it is urgent need to discover molecular biomarkers to improve the sensitivity and specificity for the detection and prognosis for cancer.

With the development of high-throughput sequencing technology, an increasing number of long non-coding RNAs (lncRNAs) have been gradually discovered and become the hotspot of research. LncRNAs, which cannot encode proteins, are important members of the non-coding RNA family. The biological functions of lncRNAs are still in its infancy and no definitive conclusion has been reached on its function and clinical significance of lncRNA. Recently, accumulating evidences have indicated that lncRNAs were closely related to initiation and progression of human diseases, especially cancer. LncRNAs could be used as a carcinogen or suppressor gene in the development and progression of cancer.

X-inactive specific transcript (XIST) is a kind of lncRNA derived from XIST gene that only expressed from the inactive X chromosome [1, 2]. Many clinical studies have clarified that the expressions of lncRNA XIST not only played an important role in the differentiation, proliferation, and genome maintenance of cells, but also with the development and progression of cancer [3]. For instance, the perturbation of lncRNA XIST expression related to metastasis and recurrence in a variety of cancers, including bladder cancer [4], nasopharyngeal carcinoma (NPC) [5], pancreatic cancer (PC) [6], colorectal cancer (CRC) [7–9], glioma [10, 11], prostate cancer (PCa) [12], ovarian cancer, gastric cancer (GC) [13, 14], hepatocellular carcinoma (HCC) [15, 16], and non-small cell lung cancer (NSCLC) [17, 18]. Nevertheless, the consistency and magnitude of the prognostic impact of lncRNA XIST remains enigmatic, and the prognostic value of lncRNA XIST expression in different tumor types remains still controversial. To verify its clinical relevance, we integrated all published evidence systematically in this meta-analysis to reveal the prognostic value of lncRNA XIST in various types of solid tumors.

## Materials and methods

### Search strategy

A systematic review of the literature was conducted according to the PRISMA guidelines. PubMed, EMBase, Web of Science, Cochrane library were searched to evaluate the impact of lncRNA XIST expression on survival in solid tumors. The following search terms included: “Long non coding RNA XIST” OR “Long Noncoding RNA XIST” OR “long non-coding RNA XIST” OR “lncRNA XIST” OR “X-inactive specific transcript” (all fields) AND “Prognosis” OR “Prognoses” OR “Prognostic” OR “Outcome” OR “survival” (all fields) AND “Neoplasia” OR “Neoplasias” OR “Neoplasm” OR “Tumor” OR “Cancer” OR “tumour” OR “carcinoma” (all fields). Moreover, the literature has been tracked to determine more relevant studies.

### Selection criteria

All collected studies were included in this meta-analysis according to the criteria as follows: (1) lncRNAncRNA XIST expression was detected only in solid tumors, not including hematologic malignancies; (2) investigation of the association between lncRNA XIST expression and survival outcome were represented in overall survival; (3) reporting sufficient data to estimate the hazard ratio (HR) and 95% confidence interval (CI) according to lncRNA XIST expression; (4) lncRNA XIST expression was detected by quantitative reverse transcription PCR (qRT-PCR) in OS tissues; (5) not a review, meta-analysis, case reports, duplicate publications.

### Data extraction and quality assessment

Data extraction of literature was as follows: first author, publication year, country of origin, cancer type, sample size, number of patients in high and low lncRNA XIST expression groups, the detection method, and the cut-off, survival analysis, the HR and 95% CI. If HR was provided in the study, we extracted them directly. Otherwise, survival data were extracted from the original study data (Kaplan–Meier curves or required data) using the software Engauge Digitizer 4.1 and calculated by Tierney. The quality of included studies was evaluated by two investigators independently according to the Newcastle–Ottawa Quality Assessment Scale (NOS). Furthermore, two investigators could resolve their differences by consensus or in discussions with a third investigator. The lowest and highest scores were scored at 0 and 9, respectively, and a study with a score greater than 6 or higher was considered a high-quality study.

### Statistical analysis

HR with 95% CI was estimated to evaluate the effective value of lncRNA XIST expression on prognosis in solid tumors. The high expression and low expression of lncRNA XIST was defined according to the cut-off values provided in the article. The heterogeneity of pooled results was evaluated using Cochran’s Q test and Higgins I-squared statistic. A statistically significant heterogeneity was defined as *p *< 0.10 or I^2^ > 50%, where a random-effect was applied. Otherwise a fixed-effect model was used. Subgroup analysis was used to further explore possible sources of heterogeneity. The stability of the results was assessed using a sensitivity analysis. The possibility of publication bias was also assessed using Begg’s test. All data were analyzed using STATA software version 12.0 (Stata Corporation, College Station, TX, USA), and a *p* value less than 0.05 was considered as statistically significant.

## Results

### Study characteristics

**Fig. 1:**
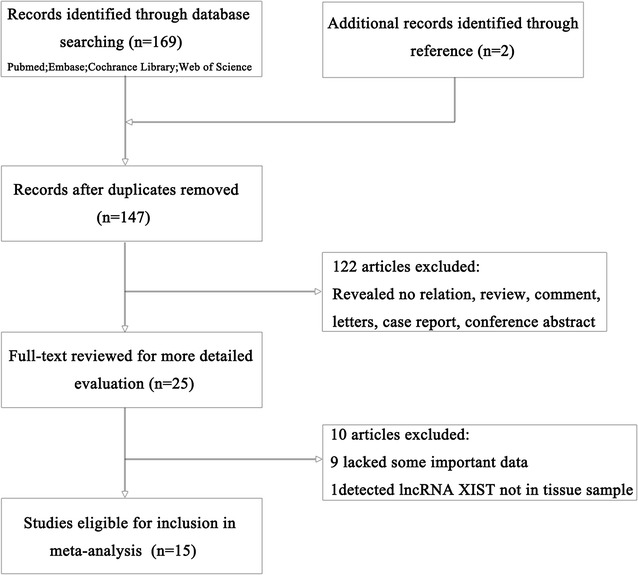
Flow diagram of the study selection process

A total of 171 related articles were retrieved, of which 169 articles were initially searched according to the criteria described in “Materials and methods” and the other 2 articles were obtained by searching the references. After screening the titles, abstracts, publication types and full text, 25 articles investigated the correlation between lncRNA XIST expression and patient survival in various tumors were selected for the systemic review. Among these, 10 articles were excluded (nine lacked some important data and one detected lncRNA XIST not in tissue sample) (Fig. 1). The total number of patients was 1209 patients from China and Japan to include in the meta-analysis, ranging from 41 to 145 patients (all considered research refers to the Asian population). The category of cancers included GC, NPC, NSCLC, HCC, cervical squamous cell carcinoma (CSCC), esophageal squamous cell carcinoma (ESCC), bladder cancer, PC, CRC, glioma, PCa, and osteosarcoma. The expression levels of lncRNA XIST was detected using qRT-PCR in all studies. OS was reported in 15 studies, while disease-free survival (DFS) had only one study. Therefore, we selected OS as the main survival outcome of all eligible studies for our meta-analysis. HR was reported directly in 6 studies and estimated indirectly in the other 9 studies. The cut-off estimates for lncRNA XIST expression was different in these studies, including the mean, median, or fold change. The detailed information about the studies was shown in Table 1.

**Table 1:** Main characteristics of all studies included in the meta-analysis

First author	Year	Country	Cancer	Total number	LncRNA XIST expression		Detection method	Cut-off (high/low)	Multivariate analysis	Survival analysis	HR and 95% CI
					High	Low					
Chen	2016	China	GC	106	54	52	qRT-PCR	4.32	No	OS	Report
Song	2016	China	NPC	108	76	32	qRT-PCR	2.31-fold	No	OS	SC
Fang	2016	China	NSCLC	53	38	15	qRT-PCR	2.58-fold	Yes	OS	Report
Ma^a^	2016	China	HCC	68	30	38	qRT-PCR	Mean	No	OS	SC
Kobayashi	2016	Japan	CSCC	49	24	25	qRT-PCR	Median	No	OS	SC
Ma^b^	2016	China	GC	98	45	53	qRT-PCR	NA	No	OS	SC
Zhang	2017	China	Osteosarcoma	41	24	17	qRT-PCR	Median	No	OS	SC
Wu	2017	China	ESCC	127	64	63	qRT-PCR	Fold change	Yes	OS	Report
Hu	2017	China	Bladder	52	32	20	qRT-PCR	Fold change	No	OS	SC
Chen	2017	China	CRC	115	58	57	qRT-PCR	Median	Yes	OS	Report
Du^c^	2017	China	Glioma	69	35	34	qRT-PCR	Median	Yes	OS	Report
Wei	2017	China	PC	64	32	32	qRT-PCR	Median	Yes	OS	Report
Du^d^	2017	China	PCa	62	37	25	qRT-PCR	Mean	No	OS	SC
Li	2017	China	Osteosarcoma	145	75	70	qRT-PCR	NA	No	OS	SC
Kong	2017	China	HCC	52	26	26	qRT-PCR	Median	No	OS	SC

HR, hazard ratio; 95% CI, 95% confidence interval; GC, gastric cancer; NPC, nasopharyngeal carcinoma; NSCLC, non-small cell lung cancer; HCC, hepatocellular carcinoma; CSCC, cervical squamous cell carcinoma; ESCC, esophageal squamous cell carcinoma; CRC, colorectal cancer; PC, pancreatic cancer; PCa, prostate cancer; qRT-PCR, quantitative reverse transcription PCR; NA, not available; OS, overall survival; SC, survival curve

^a^Weijie Ma

^b^Lei Ma

^c^Peng Du

^d^Yang Du

### Quality assessment

According to the NOS, each of the 15 eligible studies included in our meta-analysis was assessed for quality. Each one got a higher value, which indicated a better methodology. Therefore, all 15 studies were included in the subsequent analysis.

### Meta-analysis results

**Fig. 2:**
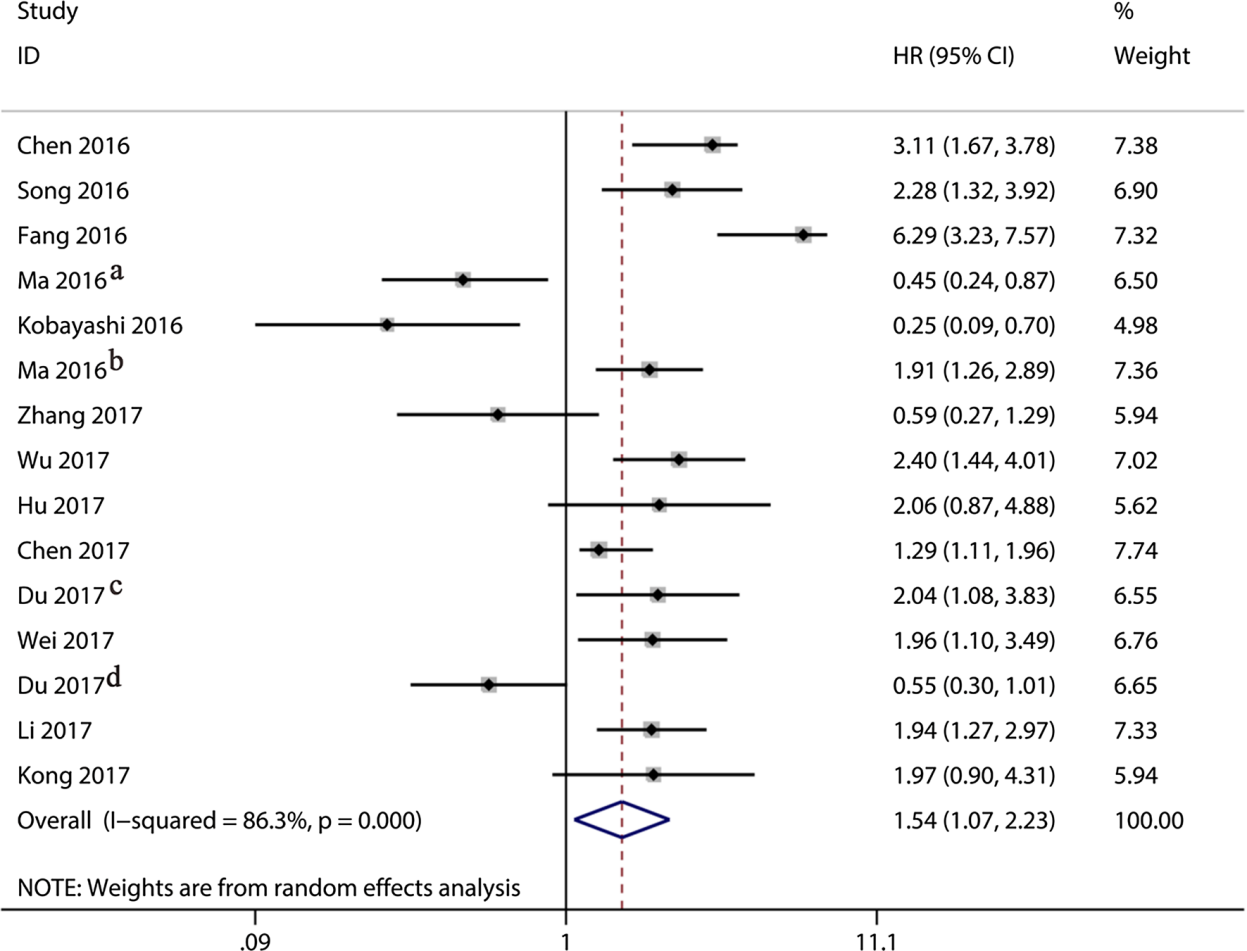
Forest plot for relationship between lncRNA XIST expression and OS. ^a^Weijie Ma; ^b^Lei Ma; ^c^Peng Du; ^d^Yang Du

The main results of the meta-analysis were shown in Table 2. The heterogeneity of 15 studies was statistically significant (I^2^ = 86.3%, *p *< 0.001), and the random-effects model was used to calculate the pooled HR and its 95% CI, which was significantly different (HR = 1.54, 95% CI 1.07–2.23, *p *= 0.021). The result showed that higher lncRNA XIST expression in cancer tissue was related to a worse OS (Fig. 2).

**Table 2:** The pooled associations between LncRNA XIST expression and the prognosis of solid tumors

Outcome subgroup	Number of patients	Number of studies	Random-effect model		Heterogeneity	
			HR (95% CI)	p value	I^2^ (%)	p value
Overall survival	1209	15	1.54 (1.07, 2.23)	0.021	86.3	< 0.001
Tumor type						
Digestive system	630	7	1.67 (1.11, 2.51)	0.014	80.5	< 0.001
Non-digestive system	579	8	1.39 (0.71, 2.74)	0.339	90.1	< 0.001
Patients’ number						
> 65	836	8	1.75 (1.24, 2.47)	0.001	78.2	< 0.001
≤ 65	373	7	1.26 (0.53, 3.01)	0.598	91.4	< 0.001
Analysis type						
Univariate analysis	781	10	1.21 (0.75, 1.95)	0.436	84.8	< 0.001
Multivariate analysis	428	5	2.39 (1.28, 4.46)	0.006	89.2	< 0.001
HR obtained method						
Reported in text	534	6	2.50 (1.49, 4.18)	0.000	87.5	< 0.001
Data extrapolated	675	9	1.07 (0.66, 1.76)	0.774	82.2	< 0.001

HR, hazard ratio; 95% CI, 95% confidence interval; OS, overall survival

Subsequently, subgroup analysis was performed to further explore the sources of heterogeneity among these studies based on four main characteristics including tumor type, patients’ number, analysis type, and HR obtained method.

In the subgroup of tumor types, a few studies such as NPC, NSCLC, bladder cancer, glioma, PCa, and osteosarcoma were collectively classified as other tumors for analysis, while more studies on the digestive system alone as a type of analysis. The OS of tumor patients with high expression of lncRNA XIST in digestive system tumors was lower than that in low expression group (HR = 1.67, 95% CI 1.11–2.51, *p *= 0.014), but not statistically significant in other tumors (HR = 1.39, 95% CI 0.71–2.74, *p *= 0.339) (Table 2).

Overall, the relationship between high lncRNA XIST expression and prolonged OS was also considered to have statistical significance in other subgroups, including patients’ number > 65 (HR = 1.75, 95% CI 1.24–2.47, *p *= 0.001), multivariate analysis (HR = 2.39, 95% CI 1.28–4.46, *p *= 0.006), reported in text (HR = 2.50, 95% CI 1.49–4.18, *p *= 0.000), respectively. However, it was not statistically significant in subgroups including patients’ number ≤ 65 (HR = 1.26, 95% CI 0.53–3.01, *p *= 0.598), univariate analysis (HR = 1.21, 95% CI 0.75–1.95, *p *= 0.436), and data extrapolated (HR = 1.07, 95% CI 0.66–1.76, *p *= 0.774). Unfortunately, there was still significant heterogeneity in these studies (I^2^ > 50%) (Table 2).

### Sensitivity analysis

**Fig. 3:**
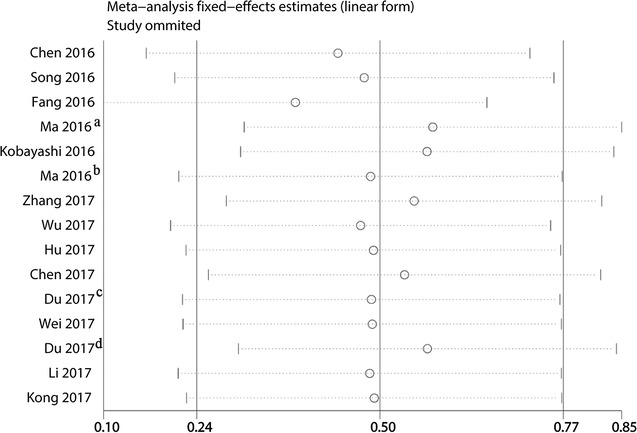
Sensitivity analysis on the relationships between lncRNA XIST expression and overall survival in solid cancer patients. ^a^Weijie Ma; ^b^Lei Ma; ^c^Peng Du; ^d^Yang Du

Sensitivity analysis was performed to evaluate the results of meta-analysis results. No significant change was found in the results when any 1 study was excluded, confirming the robustness and reliability of meta-analysis results (Fig. 3).

### Publication bias

**Fig. 4:**
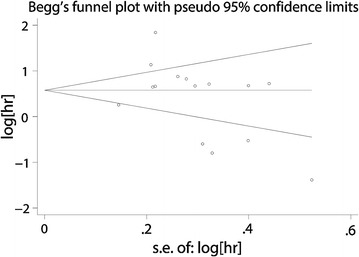
Funnel plots of publication biases on the relationships between lncRNA XIST expression and overall survival in solid cancer patients

A funnel plot, with regard to the publication bias of all studies, showed the basic symmetrical. All *p *< 0.05 (two-sided) were considered as significant. Begg’s test suggested the publication bias was not significant (Begg’s Test *p *= 0.233) (Fig. 4).

## Discussion

LncRNA XIST was a product of the XIST gene located in the X inactivation center [14], which was the first regulatory RNAs discovered to be involved in the formation of the inactive X chromosome [19]. When an X chromosome was inactivated in female animal, lncRNA XIST diffuses throughout the X chromosome, ultimately resulting in inactivation of the X chromosome [20]. Moreover, lncRNA XIST could play a dosage compensation role in female animal cells. In other words, the phenotypes determined by the gene on the X chromosome were equally expressed in XY males and XX females [15].

Aberrant expression of lncRNA XIST has been detected in many diseases. It played an important role in proliferation, migration and invasion in cancer cells in vitro and in vivo, which indicated that XIST exerted an essential role on the occurrence and development of various tumors. Differentially expressed lncRNAs could act as oncogenes or tumor suppressors to improve cancer diagnosis, discover potential treatment targets, and improve prognosis. Although many studies found that the high expression of lncRNA XIST was closely related to the prognosis of a variety of tumors, the results of the studies were quite different. It was reported that the high expression of lncRNA XIST was a risk factor for the prognosis of cancers, while some reports indicated that the high expression of lncRNA XIST was a beneficial factor in the prognosis of cancers.

For instance, Chen et al. and Wu et al. demonstrated that knockdown of lncRNA XIST suppressed cells proliferation, migration and invasion in vitro as well as tumorigenesis and metastasis in vivo in GC (2016) and ESCC (2017), respectively. Moreover, they all found that an inverse relationship between lncRNA XIST and miR-101, and knockdown of lncRNA XIST exerted its tumor-suppressive effects at least in part through regulating miR-101 to modulate EZH2 expression [14, 21]. Meanwhile, a study from Ma et al. showed that lncRNA XIST promoted cell cycle progression from the G1 phase to the S phase and protected cells from apoptosis, which contributed to GC cell growth. XIST was responsible for GC cell proliferation and invasion through the miR-497/MACC1 axis [13]. Furthermore, Temozolomide (TMZ) was the most commonly used alkylating agent in glioma chemotherapy. The data from Du et al. revealed that XIST knockdown could sensitize TMZ-resistant glioma cells to TMZ. XIST inhibited miR-29c expression by direct targeting in TMZ-resistant glioma cells [11]. In summary, it implicated that overexpression of lncRNA XIST was associated with adverse prognosis and could be used as an independent prognostic factor.

Contrary to the above tumors, increasing evidence demonstrated that XIST could also act as tumor suppressors, and played important roles in the initiation and progression of multiple cancers [12, 15, 22, 23]. For example, Kobayashi et al. observed in 2016 that the 4-year overall survival rates of patients with CSCC were 87.1 and 54.4% in the high and low XIST expression groups, respectively [22]. The results suggest that XIST could be a potential biomarker or therapeutic target for OS. However, the effect aberrant XIST expression on the prognosis of patients was still controversial in HCC and osteosarcoma. Recently, a study from Ma et al. showed that patients with JPX/XIST overexpression in HCC had longer survival times than those with low expression [15], contrary to previous research from Kong et al. [16]. Furthermore, a study from Zhang et al. revealed that lncRNA XIST regulated PDCD4 expression by interacting with miR-21-5p and inhibits osteosarcoma cell growth and metastasis [23]. While a study from Li et al. suggested that lncRNA XIST had a tumor promoter effect, and thus, to be a predictor of outcome in patients with osteosarcoma [24].

To get more accurate evidence to prove the relationship between the high expression of lncRNA XIST and the prognosis of cancers, relevant studies have been comprehensively retrieved and analyzed. Furthermore, the regulatory mechanism involved in lncRNA XIST was complex. And there was a lack of systematic research for effect of lncRNA XIST expression on tumor prognosis. Therefore, we conducted a meta-analysis to evaluate the potential value of lncRNA XIST as a novel biomarker for predicting tumor prognosis, which provided a reference for the follow-up study.

In this study, high expression of lncRNA XIST in cancer tissue was associated with poor prognosis in cancer patients (HR = 1.54, 95% CI 1.07–2.23, *p *= 0.021), with heterogeneity in the data (I^2^ > 50%). Numerous studies have shown that lncRNAs were involved in the regulation of protein-coding genes at the transcriptional and post-transcriptional levels, and it also influenced the signaling pathway pathways both intracellular as well as in organism development, thus affecting cell growth, apoptosis, and metastasis. Based on the above, deregulations of lncRNA could be a major cause of disease in human-complex diseases, including tumors. It indicated that it might serve as a negative prognostic marker for solid tumors.

Subgroup analysis and sensitivity analysis were used to investigate whether the heterogeneity of the data affected the interpretation of the analysis results. The association between lncRNA XIST overexpression and worse OS was statistically significant in digestive system tumors (HR = 1.67, 95% CI 1.11–2.51, *p *= 0.014, random-effect). These results indicated that the adverse prognostic effect of high lncRNA XIST remained substantial in digestive system cancers. In the meantime, lncRNA XIST overexpression was associated with a poor prognosis, which was statistically significant when the patients’ number is greater than 65 [patients’ number > 65 (HR = 1.75, 95% CI 1.24–2.47, *p *= 0.001, random-effect)], multivariate analysis (HR = 2.39, 95% CI 1.28–4.46, *p *= 0.006, random-effect), and reported in text (HR = 2.50, 95% CI 1.49–4.18, *p *= 0.000, random-effect).

However, there were several limitations in this paper. First of all, the different thresholds of lncRNA XIST expression were different in different studies that could not reach a uniform standard. Second, HR and 95% CI in some studies could not be obtained directly from the original literature, but HR estimates were derived from their survival curves, which might affect the results of this study. Furthermore, the limited number of included studies, all from Asians, and the small sample size (1290 cases in total) might diminish the reliability of the results. In the future, the studies of high-quality samples needed to be further confirmed.

## Conclusion

In conclusion, the high expression of lncRNA XIST was a close associate to the poor prognosis of cancer patients. LncRNA XIST overexpression might be a new unfavorable prognostic biomarker helpful for the clinical decision-making process. Considering the limitations of this analysis, this conclusion should be viewed with caution. In the future, larger sample sizes were require to confirm the prognostic value of lncRNA XIST in cancer patients and to explore more effective treatment strategies.

## Abbreviations

HR: hazard ratio
95% CI: 95% confidence interval
GC: gastric cancer
NPC: nasopharyngeal carcinoma
NSCLC: non-small cell lung cancer
HCC: hepatocellular carcinoma
CSCC: cervical squamous cell carcinoma
ESCC: esophageal squamous cell carcinoma
CRC: colorectal cancer
PC: pancreatic cancer
PCa: prostate cancer
qRT-PCR: quantitative reverse transcription PCR
NA: not available
OS: overall survival
SC: survival curve
